# Whole exome sequencing identifies a novel variant in an apoptosis-inducing factor gene associated with X-linked recessive hearing loss in a Chinese family

**DOI:** 10.1590/1678-4685-GMB-2018-0051

**Published:** 2019-11-14

**Authors:** Qi Wang, Lu Xingxing, Zhiwei Ding, Yu Qi, Yuhe Liu

**Affiliations:** 1 Department of Otolaryngology, Head and Neck Surgery, Peking University First Hospital, Beijing, China.; 2 Department of Central Laboratory, Peking University First Hospital, Beijing, China.; 3 Department of Otolaryngology-Head and Neck Surgery, The Third Affiliated Hospital, Sun Yat-sen University, Guangzhou, Guangdong, China.

**Keywords:** Whole exome sequencing, apoptosis-inducing factor, AIFM1, X-linked recessive hereditary hearing loss

## Abstract

We report on the genetic analysis of a Chinese family in which four male patients presented with postlingual progressive hearing loss, associated with distal muscle wasting and unsteady ataxic gait. Using whole exome sequencing, we identified a new pathogenic variant (c.1463C>T, p.Pro488Leu) in the *AIFM1* gene, which encodes the apoptosis-inducing factor mitochondrion-associated 1 precursor. AIFM1 is involved in the mitochondrial respiratory chain and cellular caspase-independent apoptosis pathway and has been reported to cause multiple phenotypes including hearing loss. The p.Pro488Leu missense variant segregated with symptoms in the pedigree. It was not found in the dbSNP database, databases of genomes and SNPs in the Chinese population, in 74 patients with sporadic hearing loss, or in 108 normal individuals.We also verified that this AIFM1variant enhanced cell apoptosis rates compared in 293T cells transfected with wild-type *AIFM1*. Different variations of *AIFM1* give rise to different phenotypes in patients, and this is the second reported family with a variant in the C-terminal domain of AIFM1 showing the phenotype of hearing loss and peripheral neuropathy.

## Introduction

According to different clinical manifestations, hereditary hearing loss can be divided into non-syndromic (70%) and syndromic (30%) hearing loss. Non-syndromic hearing loss (NSHL) is clinically manifested in symptoms of the auditory system alone, and is not accompanied by other organ or system abnormalities (Alford *et al.*, 2014). Syndromic hearing loss commonly involves symptoms of visual system lesions, skeletal muscle system lesions, neuropathy and neuromuscular disease, skin and metabolic diseases (Koffler *et al.*, 2015).

Mutations in the *AIFM1* (apoptosis-inducing factor mitochondrion-associated 1) gene, which encodes a flavin adenine dinucleotide-containing, NADH-dependent oxidoreductase resides in the mitochondrial intermembrane space, can cause either non-syndromic or syndromic hearing loss. Diseases caused by *AIFM1* gene mutations have previously been described as progressive mitochondrial encephalomyopathy (c.601_603delAGA, p.Arg201 deletion), Cowchock syndrome (c.1478A>T, p.Glu493Val missense mutation) or auditory neuropathy (c.1288C>T, p.Arg430Cys missense mutation) (Ghezzi *et al.*, 2010; Berger *et al.*, 2011; Rinaldi *et al.*, 2012; Kettwig *et al.*, 2015; Zong *et al.*, 2015). Although phenotype–genotype comparisons provide insights into the association between different *AIFM1* mutations and clinical manifestations, the classification of clinical characteristics caused by different mutations of *AIFM1* has hitherto remained elusive (Diodato *et al.*, 2016). In this study, using whole exome sequencing, we identified a novel variant (c.1463C>T, p.Pro488Leu) in exon 14 of the *AIFM1* gene responsible for the X-linked recessive hereditary hearing loss in a Chinese pedigree. A classification of phenotypes associated with different mutations was suggested.

## Materials and Methods

### Subjects

The study on this Chinese family with hearing loss and peripheral neuropathies (Family #36) was approved by the Medical Ethics Committee of Peking University First Hospital. All participants signed informed consent forms. Written consent for the two children who were under 18 years of age was obtained from their parents. Family investigation and physical examinations were performed in all available members by a group of otolaryngologists and audiologists. Neurological examinations included assessments of cranial nerve function, motor activity, muscle weakness, sensory impairment and reflex function. Sensorineural hearing loss diagnosis was performed in members III-5, III-9, IV-3, and IV-4 via standard audiometry according to clinical standards. Blood samples were collected from all available family members in this pedigree, as well as from 74 patients with sporadic hearing loss and 108 normal individuals used as controls at the Department of Otolaryngology and Head-Neck Surgery, Peking University First Hospital.

### Whole exome sequencing and identification of the candidate variants

Genomic DNA was extracted using the Omega E.Z.N.A.® Blood DNA Midi Kit (Omega Bio-Tek, Norcross, Georgia, USA). The samples from IV-3 and IV-4 were used to perform human exome capture, which followed the protocol from Illumina’s TruSeq Exome Enrichment Guide (Illumina, San Diego, CA, USA). Illumina’s TruSeq 62Mb Exome Enrichment kit was used as exome enrichment probe sets; 5 μg of genomic DNA in 80 μL of Elution Buffer (EB) buffer was fragmented in a biorupter to a size of 100–500 bp. DNA concentration was estimated by optical density at 260 nm (OD260) measurement and quantitative real-time polymerase chain reaction. Captured DNA libraries were sequenced with the Illumina HiSeq 2000, yielding 200 (2100) base pairs from the final library fragments using V2 reagent. Base calling was performed with casava 1.8 software (Ilumina) (Gao *et al.*, 2012). The reads were aligned with the human genome reference sequence (UCSC hg19) using the Burrows–Wheeler alignment (BWA) 0.5.9rc1. Variants (SNPs and indels) were called with vcftools of SaMTools software version 0.1.16 (Sue *et al.*, 2015).

High VarQuality SNPs were annotated with Perlscript into functional categories such as missense, nonsense, splice sites, coding, non-coding, UTRs. The probable candidate variants that were qualified by SIFT (http://sift.jcvi.org/), PolyPhen-2 (http://genetics.bwh.harvard.edu/pph2/) and MutationTaster (http://www.mutationtaster.org) were then tested by PCR (polymerase chain reaction) followed by Sanger sequencing in samples from all family members aiming to confirm segregation in this pedigree. The forward primer 5’-ATGTGCTACCGTGTCATTCCT and reverse primer 5’-AAGCCTATCAGGCCGTGTTC were used for amplification of exon 14 of the *AIFM1* gene in family members of this pedigree, 74 patients with sporadic hearing loss, and 108 normal controls. PCR products were directly sequenced using an ABI 3730 sequencer.

### Three dimensional structural model construction and mutation analysis

In order to verify the amino acid produced by nsSNPs of the *AIFM1* gene, amino acid changes on the three-dimensional structure of wild-type and mutant-type AIFM1 were constructed using Swiss Model Software (http://swissmodel.expasy.org/interactive). VMD (Visual Molecular Dynamics) software was used to analyze the three-dimensional structure modeling of wild-type and mutant-type proteins.

### Apoptosis effects of the candidate variants

A mammalian expression plasmid containing an open reading frame of human AIFM1 cDNA with a green fluorescent protein (GFP) tag at the 3’ end was obtained from Origene (Rockville, MD, USA). We used a site-directed mutagenesis kit (Transgene, Beijing, China) to produce the c.1463C>T variant in AIFM1 cDNA in the plasmids. The plasmids containing empty vector, wild-type AIFM1 or mutant-type AIFM1 were transfected into the human kidney cell line 293T cells. After 48 h, both adherent and non-adherent cells were harvested, stained with annexin-V-PE and 7-AAD (BD, Franklin Lakes, NJ, USA), and assayed in a Beckman Coulter flow cytometer. Apoptosis analysis was carried out with green fluorescent protein expression cells (GFP-positive cells). Each experiment was repeated three times. Statistical significance was determined using the independent sample *t*-test.

## Results

### Clinical features of Family #36

**Figure 1:**
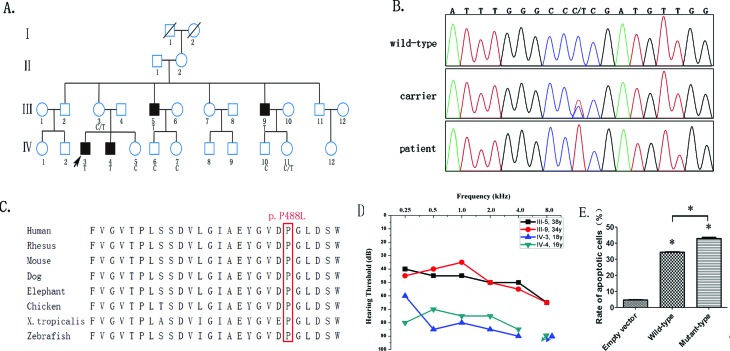
Variant analysis of AIFM1. (A) Pedigree of Family #36 demonstrates X-linked recessive inheritance hearing loss. Open symbols, unaffected; solid symbols, affected. Squares, male; circles, female; slashed, deceased individual. Slanting arrow, the proband. Family members annotated with the C symbol had no variant in AIFM1, those with the C/T symbol are carriers with the c.1463C>T (p.Pro488Leu) variant in AIFM1, those with the T symbol are patients with the c.1463C>T (p.Pro488Leu) variant in AIFM1, and those without the symbols were not examined. Segregation of hearing loss with the c.1463C>T (p.Pro488Leu) variant in AIFM1 is remarkable in this pedigree. (B) A heterozygous c.1463C>T (p.Pro488Leu) variant of the AIFM1 gene was identified in the carriers and the hemizygous variant was detected in the affected members in this pedigree. (C) Conservation analysis showed that Pro488 in human AIFM1 is conserved across human, rhesus, mouse, dog, elephant, chicken, *Xenopus tropicalis*, and zebrafish. (D) Pure tone audiometry in the four affected members of Family #36 were indicated. (E) Changes in HEK293 cells expressing c.1463C>T (p.Pro488Leu) mutant AIFM1. HEK293 cells were transfected with empty vector, wild-type AIFM1 plasmid and mutant-type AIFM1 plasmid, respectively. After transfection for 48 h, apoptotic cell ratios were determined with annexin-V-PE-staining. Data represent the mean and standard deviation of three experiments. The asterisks indicate significant differences between the control and experimental groups or differences between the wild-type group and the mutant-type group (*p<0.001).

Family #36 is a two-generation Chinese family presenting hearing loss with X-linked recessive inheritance, with four affected individuals in two generations (Figure 1A). Four male members ranging from 16 to 38 years old were diagnosed as having postlingual onset, gradually progressing, mild to severe sensorineural hearing loss involving all frequencies (Figure 1D). They did not have a history of ototoxic drug use. In the ABR (auditory brainstem response) test, there were no waveforms for each ear of the proband. The patients simultaneously presented distal muscle wasting, weakness and unsteady ataxic gait probably starting from birth and progressed slowly. The two younger affected males presented obviously more severe motor developmental disorders and there was evidence of mental retardation.

### The causative variant in Family #36

We then performed whole exome sequencing in two affected individuals (family members IV-3 and IV-4) to identify the disease causing gene in this family. The reads were first aligned with the human genome reference sequence (UCSC hg19) using the Burrows-Wheeler Alignment (BWA) tool. Variants (SNPs and indels) were called with vcftools of SAMTools software, and SNPs with a read coverage ≥ 4 and quality score ≥ 20 were considered in the initial analysis. The variants found in frequencies above 5% in the dbSNP database, yhSNP database (http://yh.genomics.org.cn/) and the 1000 Genome SNP database were excluded. A total of 1733 variants in family member IV-3, and 1679 variants in family member IV-4 were identified for further analysis. Based on the characteristics of X-linked recessive inheritance in this family, 15 shared qualified variants of family members IV-3 and IV-4 were identified in the X chromosome. The SIFT, PolyPhen-2 and MutationTaster web sites were used to predict the functional changes in the 15 candidate variants and screened out eight variants (Table 1).

**Table 1:** Eight point variants screened out in family members IV-3 and IV-4.

Chromosome	Gene symbol	Position	Ref. allele	Sample allele	Variation type	Gene region	Protein variant	Genotype	Translation impact	SIFT	PolyPhen-2
X	AIFM1	129265760	G	A	SNV	3’ UTR; Exonic	p.P488L	Het	Missense	Damaging	Probably damaging
X	FAM104B	55172537	G	A	SNV	3’ UTR; Intronic; Exonic	p.R109*	Het	Stop gain		
X	GRIPAP1	48847458	C	Insertion	Exonic	p.D121fs*57	Het	Frameshift			
X	GRIPAP1	48847459	T	C	SNV	Exonic	p.D121G	Het	Missense	Tolerated	Benign
X	HDAC6	48675847	G	Insertion	Exonic	p.I636fs*19	Het	Frameshift			
X	NHS	17705920	C	G	SNV	Exonic	p.H208	Het	Missense	Damaging	Benign
X	PRICKLE3	49034636	G	T	SNV	Exonic	p.C251*	Het	Stop gain		
X	RENBP	1.53E+08	C	A	SNV	Exonic	p.G53V	Het	Missense	Damaging	Probably damaging

Segregation of the eight variants was examined within this pedigree, and only one hemizygous variant in exon 14 of the *AIFM1* gene (c.1463C>T, p.Pro488LLeu, Fig. 1B-C) segregated perfectly with hearing loss (Figure 1A). This variant was present in hemizygosis in another two affected subjects (III-5, III-9) and was present in heterozygosis in all mothers of the affected individuals(III-3, II-2).This variant was not detected in 108 normal controls or in 74 patients with sporadic hearing loss. The proline residue at the 488th position of AIFM1 is highly conserved in most vertebrates (PolyPhen-2, http://genetics.bwh.harvard.edu/pph2/index.shtml; Figure 1D). Therefore, we considered that this novel missense variant in AIFM1 was pathogenic and responsible for the X-linked recessive hearing loss in this pedigree (Sue *et al.*, 2015).

### 3D Structural model construction and mutation analysis

**Figure 2:**
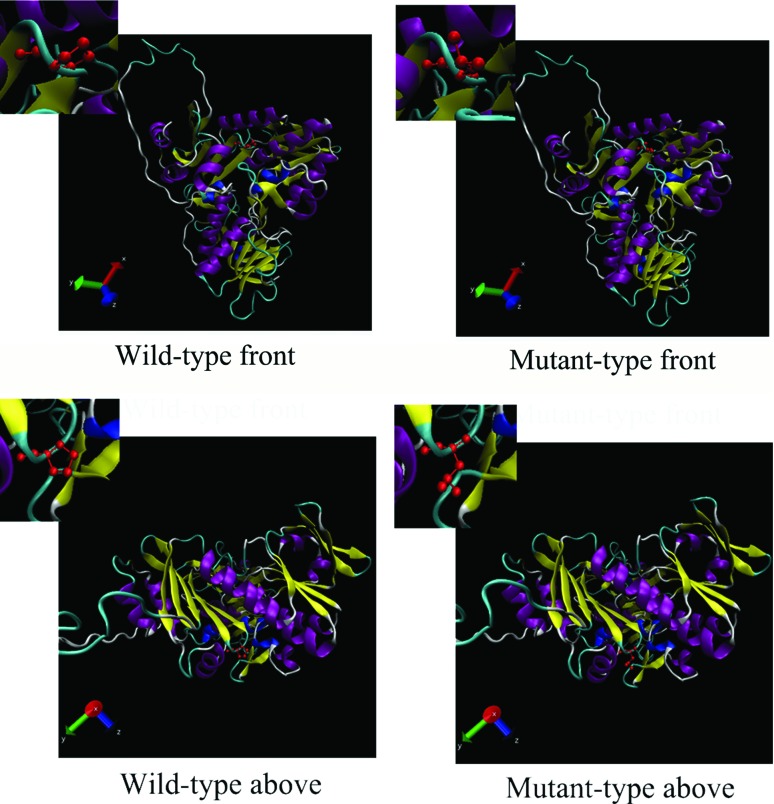
Three-dimensional structure of wild-type and mutant AIFM1. The crystal structure of mutant AIFM1 was constructed using the Swiss Model platform with the template of wild-type monomeric human AIFM1.

The crystal structure of wild-type monomeric human AIFM1 has been accurately calculated by the method of X-ray diffraction (PDB ID: 5KVI). With the template of wild-type AIFM1, the three-dimensional structure of mutant AIFM1 was constructed using the Swiss Model platform to explore the intermolecular structure and chain structure interchange. The result showed that the ring structure of this mutant site (c.1463C>T, p.Pro488Leu) is substituted by the chain structure (Figure 2).

### Apoptosis-enhancing effect in 293T cells expressing p.P488L variant AIFM1

Three groups of 293T cells were transfected with empty GFP vector, wild-type plasmid and mutant-type plasmid, respectively. After transfection for 48 hours, the cells were collected and stained with annexin V-PE and 7-AAD and assayed with flow cytometry. Successfully transfected cells, which were GFP-tag positive, were subjected to apoptosis analysis. The early and late phases of apoptotic cell rates are shown in Figure 1E. The experiments were repeated three times and the average apoptotic rates were 4.62 ± 0.57%, 34.350.58%, and 42.86 ± 1.19%, respectively. The results indicated that the p.P488L mutant AIFM1 has an apoptosis-enhancing effect in 293T cells comparing with the wild-type AIFM1 (*p*<0.001, Figure 1E).

## Discussion

AIFM1 is a flavoprotein containing three major domains: a FAD-binding domain (residues 128-262 and 401-480), a NADH-binding domain (residues 263-400), and a C-terminal domain (residues 481-608) where the apoptotic activity resides (http://atlasgeneticsoncology.org//index.html). As an NADH oxidoreductase and apoptosis inducting protein, AIFM1 responds to the fluctuations in the compartmental redox status and transmits the signal to start programmed cell death induction (Sevrioukova, 2011; Hangen *et al.*, 2015). Variant *AIFM1* that less predominantly disrupts protein production, and/or redox, and/or apoptotic inducing ability could lead to various extent of peripheral neurological disorders and/or hearing loss. Variant *AIFM1* that drastically decreased redox activity could lead to severe central neural disorders (Sevrioukova, 2016).

The typical reported AIFM1 mutations involving both clinical and molecular functional findings are summarized in Table 2. We suggest that the phenotypes of AIFM1 variants could be grouped into three types: severe-type, which includes central neural symptoms, peripheral neural symptoms and auditory neuropathy; moderate-type, which includes peripheral neural symptoms and auditory neuropathy; mild-type giving only auditory neuropathy. The family we reported here should be diagnosed as presenting the moderate type. It should be mentioned that the electrophysiological examination of peripheral nerves was not conducted in our case because of the limited instruments we could take to the remote valley where the family lived 15 years ago. This reminds us about the importance of comprehensive evaluation in patients of hereditary hearing loss families, especially those who present other symptoms.

**Table 2:** Clinical and molecular functional findings of AIFM1 mutations.

	Diagnosis	Clinical phenotypes			Molecular functions	
		Auditory neuropathy	Central neural symptoms	Peripheral neural symptoms	Mitochondrial function	Apoptosis
c. 601–603 deletion, p. Arg201del (Ghezzi *et al.,,*2010)	Mitochondrial encephalomyopathy (FAD domain)	Y	Seizures	Swallowing difficulties; dysarthria; progressive muscle weakness; decreased limb reflexes; psychomotor regression	Reduction of respiratory chain (RC) cIII and cIV	Increased apoptotic rates
c.727G>T, p. Val243Leu (Kettwig *et al.,*2015)	Ventriculomegaly + peripheral neuropathy? (FAD domain)	Y	Seizures	Swallowing difficulties; dysarthria; progressive muscle weakness; decreased limb reflexes; ataxia; psychomotor regression	NA	NA
c.923G>A, p.Gly308Glu (Berger *et al.*2011)	Prenatal ventriculomegaly (NADH domain)	NA	Seizures	Swallowing difficulties; progressive muscle weakness; psychomotor regression; cardiac involvement	NA	NA
c.1030C>T, p.Leu344Phe (Zong *et al.,*2015)	Auditory neuropathy+ peripheral neuropathy (NADH domain)	Y	N	Unsteadiness; Numbness of extremities	NA	NA
c.1463C>T p.Pro488Leu (this study)	Hearing loss + peripheral neuropathy (C-terminal domain)	Y	N	Progressive muscle weakness; decreased limb reflexes; ataxia; psychomotor regression	NA	Mildly increased apoptotic rates
c.1478A>T, p.Glu493Val (Rinaldi *et al.,,*2012)	Hearing loss + peripheral neuropathy (C-terminal domain)	Y	N	Progressive muscle weakness; decreased limb reflexes; ataxia; psychomotor regression	Slight structural changes, abnormal propensity to NADH reduction and O_2_ oxidation; faster oxidation	Higher affinity for binding to DNA and increased apoptotic rates
c.1288C>T, p.Arg430Cys (Zong *et al.,*2015)	Auditory neuropathy (FAD domain)	Y	N	N	NA	NA

The p.Pro488Leu mutation identified in this Chinese family and the previously reported p.Glu493Val mutation identified in an Italian-American family are both located in the C-terminal domain of AIFM1. The two mutations are associated to mildly increased caspase-independent apoptotic rates in cells and a varying extent of peripheral neuropathy in patients (Rinaldi *et al.*, 2012; Sevrioukova, 2016). As variations in C-terminal domain have less possibility of causing dramastic redox, they seldom result in severe neural disorders in patients. These two families also present similar phenotypes of progressive hearing loss, although further audiological tests such as speech audiometry should be carried out to clarify whether the patients of these two families present auditory neuropathy (Zong *et al.*, 2015; Moser and Starr, 2016). Auditory neuropathy is characterized by more severe impairment in speech perception (speech audiometry) compared with the performance in pure tone audiometry. These two variants in AIFM1 are likely to deteriorate the peripheral neural function including the auditory nerve, similar as the previously characterized Cowchock Syndrome (Rinaldi *et al.*, 2012).
